# Construction of a DNA Methylation Map of Argali Hybrid Sheep During Mo Infection

**DOI:** 10.3390/microorganisms14030597

**Published:** 2026-03-06

**Authors:** Qinchuan Zhang, Shiyi Li, Guojie Cheng, Guangxin Zhao, Yudie Zhou, Yanming Sun, Yanbing Zhang

**Affiliations:** College of Animal Science and Technology, Shihezi University, Shihezi 832003, China

**Keywords:** Mycoplasma ovipneumoniae, DNA methylome map, argali hybrid sheep, reduced representation bisulfite sequencing, signaling pathway

## Abstract

The DNA methylation landscape in the lungs of argali hybrid sheep infected with *Mycoplasma ovipneumoniae* (*Mo*) remains poorly characterized. This study aimed to profile genome-wide DNA methylation using reduced representation bisulfite sequencing (RRBS) and to validate key genes using bisulfite sequencing PCR (BSP), methylation-specific PCR (MSP), and quantitative MSP (QMSP). The results revealed a significant increase in global mCG methylation in -infected lungs. RRBS identified 3691 differentially methylated regions (DMRs), 66.2% of which were hypermethylated. Methylation levels were highest in gene bodies/downstream regions and lowest in promoters/5′ untranslated regions. Differentially methylated genes (DMGs) were enriched in immune–inflammatory pathways (e.g., antigen presentation, B-cell receptor signaling, Th17 differentiation) and, to a lesser extent, neural signaling pathways. BSP confirmed the methylation status of hypermethylated *(KHDC3L*, *GILT*, *OVAR-DRB1*, *SGK1*, *ADAM17)* and hypomethylated (*EFCAB11*, *AP1B1*, *TATDN1*) DMGs. Independent validation by MSP and QMSP further supported the hypermethylation of *SGK1* and *GILT* in both lung tissue and alveolar macrophages. Quantitative reverse-transcription PCR showed that promoter hypermethylation of *KHDC3L*, *GILT*, *SGK1*, and *ADAM17* was associated with transcriptional downregulation, while hypomethylation of *AP1B1* correlated with upregulation. In summary, *Mo* infection induces genome-wide hypermethylation reprogramming that dysregulates key immune-related genes, highlighting potential epigenetic mechanisms in the pathogenesis of mycoplasmal pneumonia.

## 1. Introduction

*Mycoplasma ovipneumoniae* (*Mo*) is a primary etiological agent of mycoplasmal pneumonia in sheep, goats, and wild argali. The infection presents a range of clinical signs, from subclinical infections to typical pneumonia. The key characteristics of the disease in the infected sheep and goats include paroxysmal coughing, wheezing, progressive wasting, and interstitial proliferative pneumonia. Critically, *Mo* infection is a key predisposing factor for secondary bacterial infections, and such co-infections significantly elevate the mortality rates in sheep [1]. In recent years, recurrent outbreaks of *Mo* infection have intensified worldwide, with *Mo*-related pneumonia reported in multiple regions, including Savannakhet Province, Laos, and Qinghai, China [2,3], resulting in significant economic losses to the sheep farming industry. Currently, effective control of *Mo* infection is hampered by the lack of both high-efficacy vaccines and targeted therapeutics. Although previous studies have characterized *Mo* virulence factors [4] and host inflammatory responses [5,6], the molecular mechanisms underlying *Mo*-induced immune suppression and host regulatory dysregulation remain largely unresolved. Crucially, the molecular pathways by which DNA methylation regulates immune responses and drives tissue injury have not yet been systematically elucidated.

DNA methylation dynamically modulates genomic transcription activity [7,8,9]. During infection, pathogens drive the reprogramming of host genomic methylation patterns, leading to dysregulation of key immune functions such as inflammatory signaling, antigen presentation, and apoptosis [10]. Hypermethylation often results in the downregulation or even silencing of expression of immune-regulatory genes, especially those involved in anti-inflammatory responses and immune checkpoint pathways [11,12]. In contrast, hypomethylation can promote the upregulation of expression of proto-oncogenes and inflammatory mediators [13]. Notably, *Mycobacterium tuberculosis* infection induces hypermethylation of the interleukin-12B (IL-12B) promoter in host macrophages, leading to the downregulation of expression of Th1-type immunity [14], whereas hepatitis B virus (HBV) promotes proto-oncogene hypomethylation by downregulating DNMT1 methyltransferase activity [15]. Together, these findings highlight the pathogen-mediated reprogramming of host DNA methylomes. However, the epigenetic consequences of *Mycoplasma* infection remain largely uncharacterized in ruminant species.

Reduced representation bisulfite sequencing (RRBS) has emerged as a powerful method for genome-wide DNA methylation profiling, offering high sensitivity, cost-effectiveness, and single-base resolution [16,17]. Unlike genome-wide bisulfite sequencing, RRBS uses *Msp*I digestion to selectively enrich CpG-dense regions, thereby enabling coverage of >85% of promoters and CpG islands (CGIs) at substantially reduced costs [18]. Although RRBS has been successfully applied to epigenetic investigations of BVDV [19] and *Piscirickettsia salmonis* infections [20], a comprehensive characterization of the host DNA methylome map during *Mycoplasma* infection remains to be established.

In this study, RRBS technology was employed to profile the genome-wide DNA methylome of lung tissue from *Mo*-infected argali hybrid sheep. The study aimed to: (1) describe the global alterations in DNA methylation levels associated with *Mo* infection; (2) identify and functionally characterize differentially methylated regions (DMRs) and their related genes (DMGs), with a focus on their potential involvement in immune regulation and metabolic processes; and (3) explore the correlation between promoter methylation status and the expression of key candidate DMGs. As a foundational descriptive study, these findings provide a preliminary epigenetic landscape and candidate loci, which may inform future mechanistic investigations into the pathogenesis of *Mo* infection.

## 2. Materials and Methods

### 2.1. Mycoplasma Strain, Culture, and Experimental Materials

The Mycoplasma ovipneumoniae strain XJ-F55 (Mo-XJ-F55), isolated from infected sheep lungs in Shihezi, China (2021), was used in this study and cryopreserved at −80 °C. The mycoplasma was cultured in PPLO broth (without crystal violet) supplemented with horse serum.

The pUCm-T vector and DH5α competent cells were obtained from Sangon Biotech (Shanghai, China). Tissue DNA was extracted using the Vazyme DNA Isolation Mini Kit (Nanjing, China; Cat# DC-103-01). For bisulfite conversion, the EpiJET Bisulfite Conversion Kit (Thermo Fisher Scientific, MA, USA; Cat# 2922819) was employed. Total RNA was extracted with TRIzol reagent (Invitrogen, CA, USA) and reverse-transcribed using the HiScript IV RT SuperMix for qPCR (+gDNA wiper) from Vazyme (Cat# R423-01).

### 2.2. Experimental Design and Sample Collection

**Figure 1:**
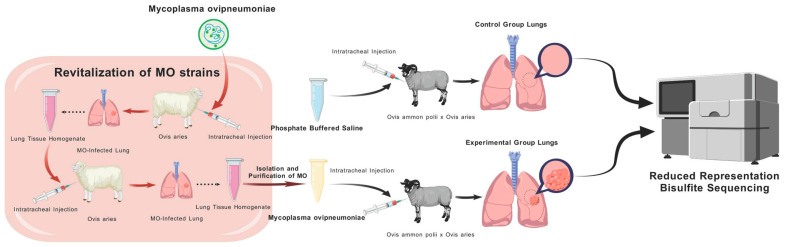
Flowchart of the experimental design process for establishing an *Mo* infection model in hybrid argali sheep, collecting qualified lung tissue samples, and performing RRBS analysis. The diagram was created using BioGDP.

In this study, an *Mo* infection model was established using argali hybrid sheep. The lung tissue samples were then collected from both the *Mo*-infected and control groups. RRBS was performed to investigate genome-wide DNA methylation changes triggered by *Mo* infection. The specific experimental design is shown in Figure 1.

Following the revival of the *Mo* strain Mo-XJ-F55, a suspension containing 1 × 10^8^ CCU/mL was administered intratracheally to male Hu sheep (Ovis aries; 3 months old, 20.1 ± 0.5 kg) after an acclimatization period of 15 days. At 21 days post inoculation (dpi), lung tissues were aseptically collected from the margins of gross pathological lesions, specifically at the interface between hepatized and healthy lung tissues. Tissues were homogenized in ice-cold phosphate-buffered saline (PBS; 1:5 *w*/*v*) using liquid nitrogen-cooled mortar and filtered through a triple-layer sterile gauze to obtain primary inoculum (F0 homogenate). For pathogen amplification, 5 mL of F0 homogenate (containing 1.9 × 10^5^ copies/μL, quantified by HSP70 qPCR) was inoculated intratracheally into a second SPF Hu sheep. Identical processing at 21 dpi yielded amplified inoculum (F1 homogenate). Six male argali hybrid sheep (3 months old, weighing 22.4 ± 0.5 kg), adapted to captivity, were randomly divided into two groups: the *Mo* group (*n* = 3) received a tracheal injection of fresh inoculum (F1 homogenate) (1.9 × 10^5^ copies/μL), whereas the control group (*n* = 3) received an equal volume of sterile PBS. At 21 dpi, the sheep were anesthetized and euthanized. Lung tissue was collected under aseptic conditions. From each animal in the *Mo* group, 50 mg of tissue was collected from the lesion margin between hepatized and healthy lung tissues. For each sheep in the control group, 50 mg of normal tissue was collected from the corresponding anatomical site. All six samples were immediately frozen in liquid nitrogen and sent to Novogene Co., Ltd. (Beijing, China) for sequencing.

### 2.3. DNA Extraction, Preparation of RRBS Libraries and Methylation Extraction

Genome-wide DNA methylation profiling using RRBS was performed on six pulmonary tissue specimens (3 samples of *Mo* group as *Mo* 1, *Mo* 2, *Mo* 3 replicates, and 3 samples of control group as control 1, control 2, control 3 replicates), and sequencing was conducted by Novogene Co., Ltd. (Nanjing, China). Following successful verification of the DNA quality, samples underwent enzymatic digestion using the methylation-insensitive restriction endonuclease *Msp*I to generate fragments of 40–220 bp. The fragments were then subjected to end repair, 3′-terminal adenylation, and ligation with comprehensively cytosine-methylated Illumina sequencing adapters to minimize PCR amplification bias. The ligation products were subjected to bisulfite conversion using the EZ DNA Methylation-Gold Kit (Cat: D5005; Zymo Research, CA, USA), facilitating the deamination of unmethylated cytosines to uracils. The converted DNA templates were then PCR-amplified and purified using the QIAquick Gel Extraction System (Qiagen, NY, USA). The final library sequencing was performed on the Illumina NovaSeq 6000 platform with 150 bp paired-end reads. The raw sequencing reads were subjected to stringent quality filtration to remove adapter contaminants and low-quality bases (Phred score < 30), yielding high-fidelity clean datasets. The clean reads were aligned to the reference genome using Bismark’s methylation extractor module (v0.24.0) for comprehensive cytosine methylation analysis. Detailed sequencing metrics for all specimens are presented in Table 1.

### 2.4. Extraction of Differentially Methylated Regions

Differential methylation analysis in *Mo* group with respect to control group was performed using the DSS package (v2.12.0) [21] within the RStudio environment (4.5.1 version) to identify differentially methylated loci (DML) [22]. Statistically significant DML were filtered using the callDML function, applying a false discovery rate (FDR) threshold of <0.05 and an absolute methylation difference cutoff (|Δ*β*| ≥ 0.1). These DML were subsequently used to define DMRs [23]. The candidate regions were initially demarcated through a sliding window approach requiring a minimum coverage of five CpG sites per window. Adjacent DMRs were iteratively merged only if the following criteria were met: (1) the continuous genomic segment, from the start of the upstream DMR to the end of the downstream DMR, exhibited a ≥2-fold intergroup methylation difference; and (2) Fisher’s exact test reached statistical significance (*p* < 0.05). Conforming regions were consolidated into unified DMRs, whereas nonconforming intervals remained as independent DMRs, producing the final DMR dataset after convergence. The genomic distributions and statistical significance of the identified DMRs were visualized using Circos (v0.62-1). Methylation levels are calculated using the methylKit R package (1.32.1 version).

### 2.5. Extraction of Differentially Methylated Genes and Their Functional Annotation

Functional enrichment analysis was conducted for genes overlapping DMRs within gene bodies [defined as regions spanning the transcription start site (TSS) to transcript end site (TES)] based on the genomic distribution patterns of DMRs. Gene Ontology (GO) enrichment analysis was conducted using the Goseq package [24], which corrects for gene length–dependent bias. Fisher’s exact test was used to identify significantly enriched terms across the three GO categories: biological processes, cellular components, and molecular functions. The enrichment results were visualized as bar charts, with GO annotations sourced from http://www.geneontology.org (accessed on July 17 2025). Further, the Kyoto Encyclopedia of Genes and Genomes (KEGG) pathway enrichment analysis [25] was performed using KOBAS 2.0 to identify pathways significantly enriched with DMGs relative to the genomic background. Significance was assessed with Fisher’s exact test using KEGG pathways as functional units. The results were visualized as bubble plots. Enrichment significance was evaluated using three integrated metrics: enrichment factor (ratio of enriched DMGs to total annotated genes per pathway), *q*-value (FDR-adjusted *p* value), and the count of enriched DMGs. For both GO and KEGG analyses, terms were considered significant only if Fisher’s exact test *p* value was <0.05 and *q*-value < 0.05. Identical analytical procedures were applied to DMGs located within promoter regions.

### 2.6. Validation of DMGs Using Bisulfite Sequencing PCR

Genomic DNA was isolated from lung tissues using the DNA Isolation Mini Kit, with concentration quantified using a NanoDrop One spectrophotometer (Thermo Fisher Scientific, MA, USA). Bisulfite conversion of 400 ng/μL DNA aliquots was performed using the EpiJET Bisulfite Conversion Kit. Bisulfite sequencing PCR (BSP) primers were designed using MethPrimer. The converted DNA templates were amplified with BioGold 2 × SuperPCR Master Mix (containing Super Pfx DNA Polymerase). The amplification products were electrophoresed on agarose gels, and target fragments were excised for sequencing (Sangon Biotech, Shanghai, China). The sequence-verified fragments were cloned into pUCm-T vectors, and the ligation products were transformed into competent cells and plated on ampicillin-containing LB agar for overnight incubation at 37 °C.

For each target region, approximately 30 positive clones (15 per *Mo*-infected/control group) were Sanger-sequenced using M13 primers. The non-CpG C-T conversion efficiency was evaluated using the quantification tool for methylation analysis (QUMA) platform (http://quma.cdb.riken.jp) (accessed on July 20 2025). The methylation levels were calculated as the percentage of methylated CpG sites across all analyzed clones. The intergroup methylation differences were assessed using Fisher’s exact test. The gene-specific primer sequences are provided in the Supplementary Materials, Table S1.

### 2.7. Validation of Methylation Level of DMGs Using Methylation-Specific PCR and Quantitative Methylation-Specific PCR

Total DNA was extracted from lung tissue samples and alveolar macrophages (AMs) of both the *Mo* and Control groups using a commercial DNA extraction kit. The DNA concentration was normalized to 500 ng/μL, followed by bisulfite conversion using the EpiJET Bisulfite Conversion Kit. Primers for methylation-specific PCR (MSP) and quantitative MSP (QMSP) were designed on the MethPrimer website, and their sequences are provided in the Supplementary Materials, Tables S1 and S2.

Bisulfite-converted DNA from lung tissue was amplified using BioGold 2 × SuperPCR Master Mix (containing Super Pfx DNA polymerase) under the following MSP conditions: initial denaturation at 95 °C for 3 min; 35 cycles of 95 °C for 10 s, 58 °C for 30 s, and 72 °C for 20 s; final extension at 72 °C for 3 min; hold at 4 °C. The PCR products were separated on 1.5% agarose gels, and the band fluorescence intensity was quantified with ImageJ (1.54f) software to assess the methylation level of the target gene.

For quantitative methylation-specific PCR (QMSP), bisulfite-converted DNA from lung tissue and AMs was mixed with BioGold SYBR qPCR Master Mix (SYBR Green I dye). Sheep β-actin was used as the endogenous control. Amplification was performed on a LightCycler 96 system (Roche) under the following program: initial denaturation at 95 °C for 180 s; 40 cycles of 95 °C for 5 s and 58 °C for 20 s; followed by a melting curve analysis (95 °C for 15 s → 60 °C for 60 s → 95 °C for 15 s). The methylation percentage was calculated using the formula:

$$Methylation\;(\%)=\frac{100}{1+2^{CTCG-CTTG}}$$

where CT_CG and CT_TG represent the threshold cycle values for the methylated and unmethylated states, respectively [26]. This formula was applied to determine the DNA methylation levels of the target gene in both the *Mo* and Control groups.

### 2.8. Validation of Differential Expression of DMGs Using RT-qPCR

Total RNA was extracted from the lung tissues of sheep in the *Mo*-infected and control groups using TRIzol reagent, strictly following manufacturer’s protocols. RNA concentration and purity were assessed using the NanoDrop One spectrophotometer. The samples were normalized to 1000 ng/μL total RNA concentration, with 2 μL of RNA used for cDNA synthesis with HiScript IV RT SuperMix for qPCR (+gDNA Wiper) to eliminate genomic DNA. The qPCR reactions contained cDNA template mixed with BioGold SYBR qPCR Master Mix (SYBR Green I dye), using sheep β-actin as the endogenous control. Amplification was performed on a LightCycler 96 system (Roche) under the following cyclic conditions: initial denaturation at 95 °C for 180 s, 40 cycles of 95 °C for 5 s and 54 °C for 20 s; followed by a melting curve of 95 °C for 15 s → 60 °C for 60 s → 95 °C for 15 s. Relative gene expression levels were calculated using the 2^−ΔΔCt^ method. The gene-specific primer sequences are provided in the Supplementary Materials, Table S2.

### 2.9. Statistical Analysis

BSP data were processed using the QUMA online analysis platform (http://quma.cdb.riken.jp) (accessed on July 20 2025), and the differences in methylation levels were assessed using Fisher’s exact test. Reverse transcription (RT)-qPCR data were analyzed using GraphPad Prism 8.0 software (GraphPad Software, CA, USA). All data were presented as the mean ± standard deviation of three independent experiments. Intergroup comparisons were performed using Student’s *t* test based on the data type. Statistical significance was determined using the following criteria: * *p* < 0.05, ** *p* < 0.01, and *** *p* < 0.001.

## 3. Results

### 3.1. RRBS Libraries, Extracted Methylated Loci and Their Genomic Localization

After rigorous quality control filtering, an average of 34,649,532 high-quality clean reads (Q30 > 91.07%, bisulfite conversion rate > 99.5%) was obtained per sample. The data have been deposited in the NCBI database (BioProject ID: PRJNA1255582).

**Figure 2:**
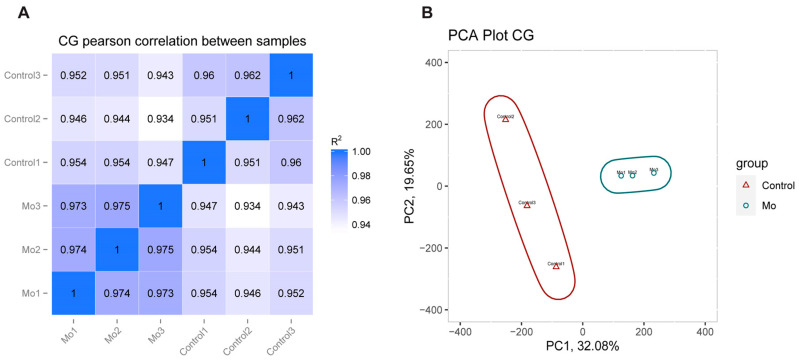
Multi-method analysis showing intergroup and intragroup similarities and differences in genome-wide methylation patterns between control groups and *Mo* groups. (**A**) Pearson correlation matrix of CG methylation patterns. Axes represent individual samples; R^2^ indicates the squared Pearson correlation coefficient. Square shading denotes methylation pattern similarity, with darker shades reflecting higher correlation coefficients (closer to 1). (**B**) Principal component analysis of methylation profiles. Axes represent principal components 1 (PC1) and 2 (PC2), accounting for maximal inter-sample variance. Confidence ellipses denote group distributions: red triangles represent the control group (*n* = 3), and blue circles represent the *Mo*-infected group (*n* = 3). Increased separation between ellipse centroids indicates stronger inter-group differentiation. Smaller ellipses reflect lower data dispersion and higher within-group homogeneity.

We successfully established a *Mo* infection model in hybrid argali sheep and collected qualified lung tissue samples for RRBS sequencing. Focusing on the dominant mCG methylation pattern, the analysis revealed high concordance of CG methylation among samples within the same group (Figure 2A), but significant differences between the *Mo*-infected and control groups (Figure 2B).

**Figure 3:**
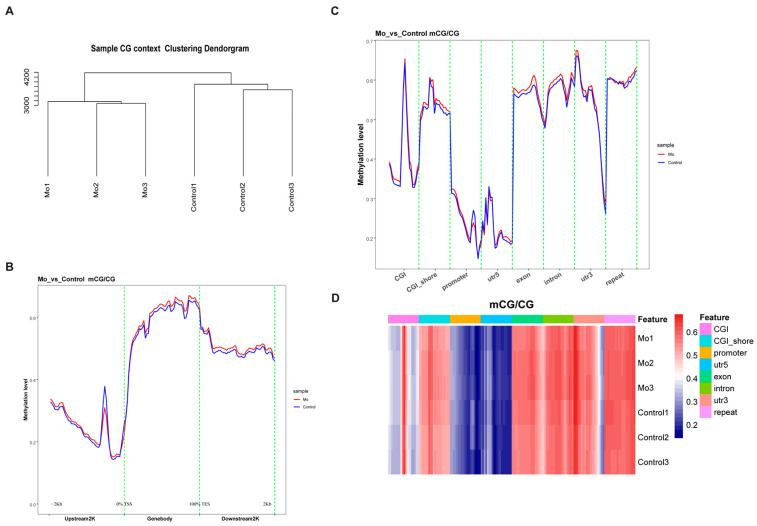
Comparative analysis of genome-wide DNA methylation profiles between *Mo*-infected and control groups. (**A**) Cluster dendrogram based on CG methylation similarity (Bray–Curtis index). The horizontal axis represents individual samples, and the vertical axis indicates the distance between clusters, where longer branches correspond to lower similarity (i.e., greater dissimilarity) between samples. (**B**) Methylation density distribution across gene regulatory regions. Each region—including the upstream 2 kb promoter, gene body, and downstream 2 kb terminator—was divided into 50 bins. The heatmap shows the mean CG methylation density (β value) per bin for the *Mo* group (red) and control group (blue). (**C**) Methylation profiles of core genomic elements. Functional elements were partitioned into 20 bins, and the heatmap displays the average CG methylation density per bin in *Mo*-infected (red) versus control (blue) samples. (**D**) Site-resolved methylation heatmap of functional genomic regions. The top color bar indicates annotations of genomic elements. Each tile represents the β value of an individual CG site (blue: hypomethylated; red: hypermethylated).

Clustering analysis based on CG methylation patterns revealed clear intergroup separation: samples from the *Mo* and Control groups clustered separately, with high similarity in methylation profiles within each group and considerable differences between the two groups (Figure 3A). Systematic construction of a DNA methylome map indicated that the gene body region exhibited the highest average methylation level (ML = 0.61), followed by the downstream 2 kb region (ML = 0.51), whereas the upstream 2 kb region displayed the lowest level (ML = 0.25) (Figure 3B). A detailed analysis of genomic localization of extracted methylated loci demonstrated significantly lower methylation levels in the promoter (ML = 0.22) and 5′ untranslated regions (5′UTR; ML = 0.28). In contrast, higher methylation levels were observed in CGI (ML = 0.44), CGI shore (ML = 0.54), exon (ML = 0.55), intron (ML = 0.56), 3′UTR (ML = 0.51), and repeat regions (ML = 0.61) (Figure 3C). Heatmap analysis further confirmed the characteristic low methylation in promoter and 5′UTR regions (Figure 3D).

**Figure 4:**
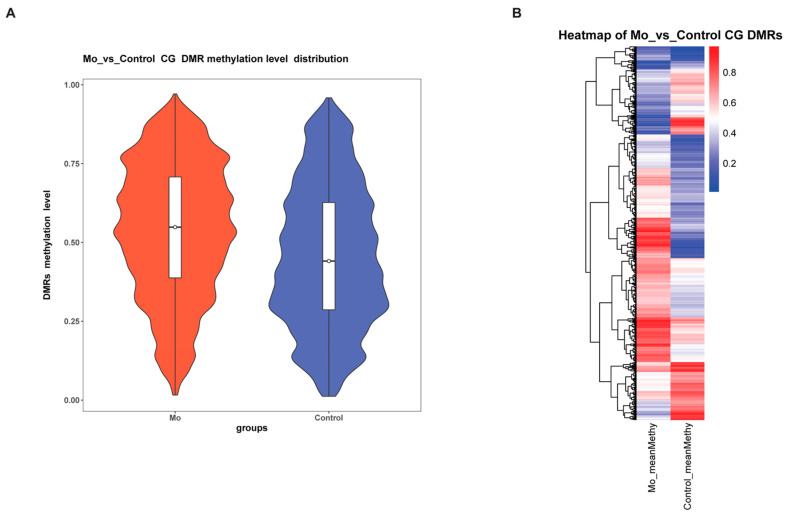
*Mo* infection increased global methylation levels in argali hybrid sheep. (**A**) Distribution of DMR. Violin plots display β-value distributions of all DMRs (boxplots: interquartile ranges; contours: kernel density estimation), and red is the MO-infect group, blue is the Control group. (**B**) Hierarchical clustering analysis of DMR methylation patterns. Rows correspond to differentially methylated regions clustered by Euclidean distance, and columns represent the experimental groups. Tile colors reflect the gradient of β-values, with blue to red indicating low–high methylation levels, respectively. The height of the dendrogram branches is proportional to the degree of dissimilarity.

DMR analysis showed that the overall methylation level in the *Mo*-infected group (average ML = 0.54) was significantly higher than that in the control group (average ML = 0.43) (Figure 4A). The DMR clustering heatmap (Figure 4B) identified eight major clusters of methylation differences, revealing a predominance of DMR hypermethylation in the *Mo*-infected group.

In summary, *Mo* infection induced significant alterations in genome-wide mCG methylation levels in the lung tissue of argali hybrid sheep, demonstrating an overall hypermethylation trend.

### 3.2. Differentially Methylated Regions and Genes in Mo-Infected Argali Hybrid Sheep

**Figure 5:**
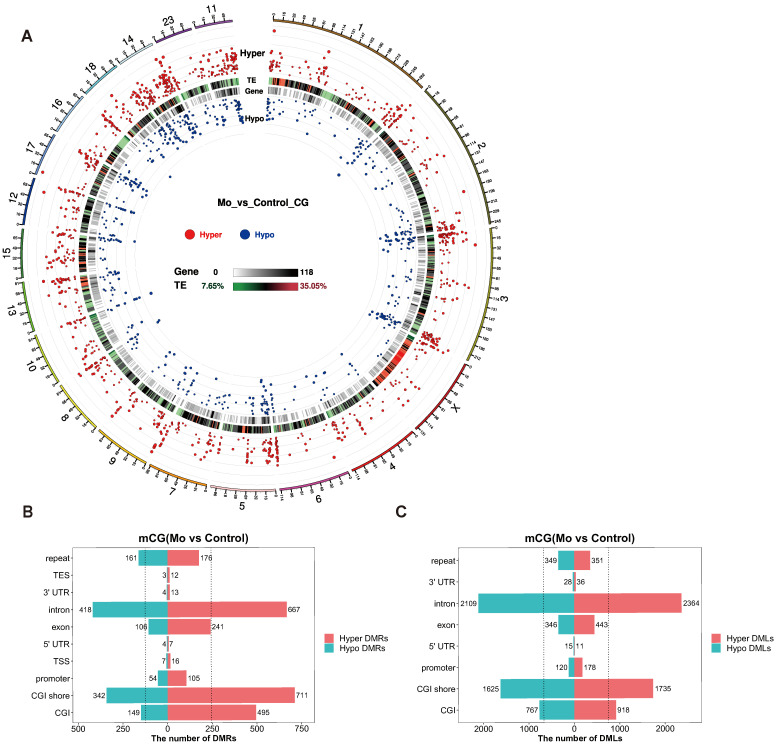
Genome-wide DNA methylation landscape construction. (**A**) Circos plot illustrating DMR-associated CpG sites. Tracks from periphery to center: (1) chromosomal map based on the reference genome of argali hybrid sheep (Ovis ammon polii × Ovis aries); (2) hypermethylated DMR statistical value, shown as log_5_(|areaStat|); radially outward dot height proportional to differential methylation significance (red dots); (3) transposable element (TE) density; color gradient indicates TE density (red: high; green: low); (4) gene density heatmap, with grayscale intensity corresponding to gene density (black, high; white, low); and (5) hypomethylated DMR statistical value, shown as log_5_(|areaStat|); radially inward dot height proportional to differential methylation significance (blue dots). (**B**) Genomic distribution of DMR-anchored regions. The horizontal axis represents the number of hyper- and hypomethylated DMRs in each region, and the vertical axis represents the region categories; the dotted line represents the average total number of hyper-and hypomethylated DMRs. (**C**) Genomic distribution of DML-anchored regions. The horizontal axis represents the number of hyper- and hypomethylated DML in each region, and the vertical axis represents the region categories; the dotted line denotes the average total number of hyper- and hypomethylated DML.

Within the mCG methylome, we identified 3691 DMRs, including 2443 hypermethylated DMRs and 1248 hypomethylated DMRs. To visualize their genome-wide distribution, we constructed a DNA methylome map using the Circos framework (Figure 5A). In this map, red dots extending outward and blue dots extending inward denote the locations and statistical significance (log5(|areaStat|)) of hyper- and hypomethylated DMRs, respectively. Each radial line along the chromosome corresponds to a DMR, with the relative abundance of hyper/hypomethylated regions reflecting the overall methylation levels at that position. The map also includes the distribution of transposable elements (TEs), highlighting that regions with high TE density may be associated with epigenetic silencing [27]. Further analysis segmented DMRs across genomic features, including CGI, CGI shore, promoter, TSS, 5′ UTR, exon, intron, 3′ UTR, TES, and repeat sequences (Figure 5B). Statistical analysis of the 3691 DMRs, along with 11,395 DML within the CGI, CGI shore, promoter, 5′ UTR, exon, intron, 3′ UTR, and repeat regions (Figure 5C, including 6036 Hyper DML and 5359 Hypo DML), revealed a consistent trend. The number of hypermethylated DMRs/DML exceeded that of hypomethylated DMRs/DML across all analyzed regions. Collectively, these results demonstrated that *Mo* infection significantly elevated DNA methylation levels at both genome-wide and chromosomal levels, indicating that hypermethylation across diverse genomic regions is a key characteristic of the infection response.

### 3.3. GO Analysis of DMGs

Our GO enrichment analysis of differentially methylated genes (DMGs) identified a total of 2335 significant GO terms. These comprised 1388 biological processes, 331 cellular components, and 616 molecular functions.

**Figure 6:**
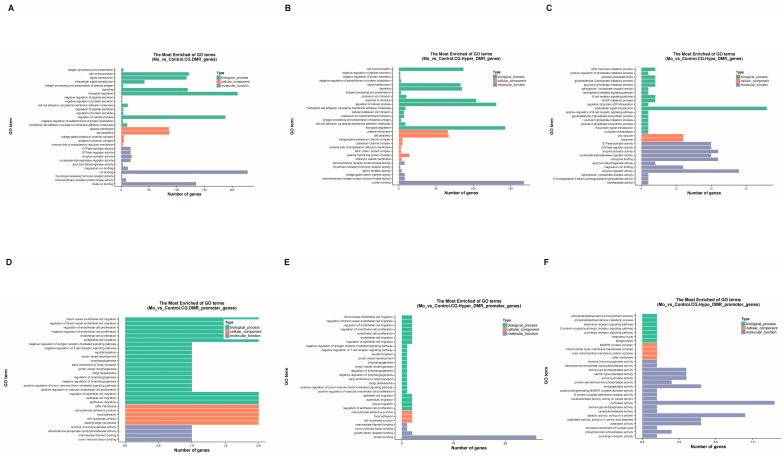
Presents a comprehensive Gene Ontology (GO) enrichment analysis of differentially methylated genes (DMGs). Panels A–C display the results for all identified DMGs: global enrichment (**A**), hypermethylated DMGs (**B**), and hypomethylated DMGs (**C**). Panels (**D**–**F**) focus specifically on DMGs located in promoter regions, showing the corresponding enrichment for all (**D**), hypermethylated (**E**), and hypomethylated (**F**) promoter DMGs. In all panels, the vertical axis lists the top 20 significantly enriched GO terms (*p* < 0.05), and the horizontal axis indicates the number of genes associated with each term. Bars are color-coded by GO category: biological process (green), cellular component (coral), and molecular function (steel blue).

When focusing on significant biological processes (*p* < 0.01), we found that genes associated with all DMRs were notably enriched in immune regulatory functions. The terms “biological regulation” (210 genes) and “regulation of cellular process” (188 genes) contained the largest number of genes. However, the term “antigen processing and presentation of peptide antigen via MHC class I” showed the highest enrichment significance (Figure 6A). This underscores a strong link to adaptive immune responses.

We next analyzed hypermethylated and hypomethylated DMGs separately. Hypermethylated DMGs were associated with 1157 biological process terms. They were primarily enriched in broad regulatory categories, including “biological regulation” (143 genes) and “regulation of cellular process” (131 genes). Among these, “cell communication” (87 genes) displayed the strongest enrichment (Figure 6B and Figure S1A). This pattern highlights a potential widespread role in modulating cellular signaling and coordination. In contrast, hypomethylated DMGs corresponded to 856 biological process terms. They showed specific enrichment in more defined functional areas. The most notable terms were “intracellular signal transduction” (18 genes) and “GDP–mannose metabolic process,” which exhibited the highest enrichment score (Figure 6C and Figure S1B). This suggests a primary involvement in signal transduction and specific metabolic pathways.

We also performed a focused analysis on DMGs located within promoter regions. These promoter DMGs were linked to 965 GO terms (621 biological processes, 150 cellular components, 194 molecular functions). For significantly enriched biological processes (*p* < 0.01), most terms were related to signal pathway regulation and cellular phenotype transformation. Specifically, “blood vessel endothelial cell migration” and its related regulatory terms showed the highest enrichment significance (Figure 6D). This finding may be relevant to vascular changes during lung inflammation. Promoter-specific hypermethylated DMGs (568 biological process terms) showed a significant peak for terms associated with vascular endothelial cell migration (Figure 6E). This points to their potential role in processes involving vascular remodeling and signaling networks. Conversely, promoter-specific hypomethylated DMGs (160 biological process terms) were uniquely enriched in the “phosphatidylethanolamine biosynthetic process” (Figure 6F). This enrichment peak suggests a specific link to membrane structure dynamics and the regulation of autophagy.

In summary, our GO analysis reveals a clear functional distinction. Hypermethylated DMGs are predominantly involved in overarching biological regulation and signal integration. Hypomethylated DMGs are more specifically engaged in discrete signal transduction and metabolic processes. The promoter-specific analysis further refines this view. It links promoter hypermethylation to pathways like vascular migration, while promoter hypomethylation is tied to membrane biosynthesis and autophagy. Together, these results systematically outline the functional specificity of DNA methylation changes within key cellular networks during infection.

### 3.4. KEGG Analysis of DMGs

**Figure 7:**
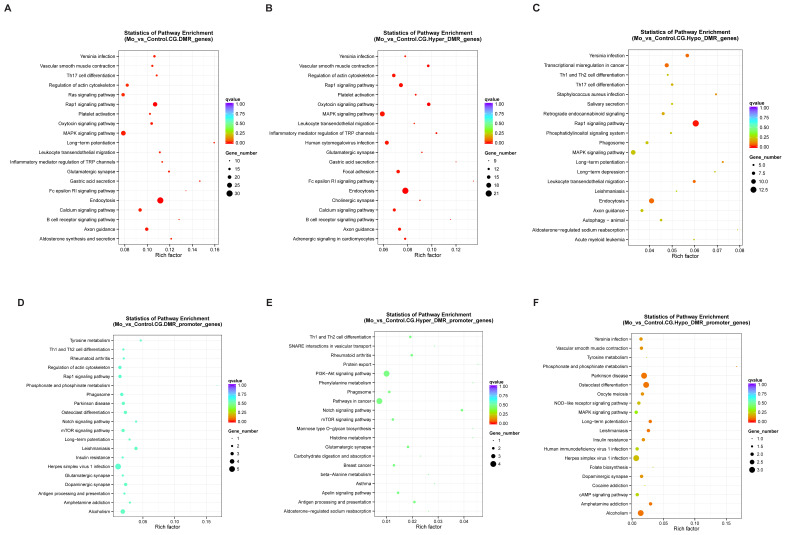
Presents the KEGG pathway enrichment analysis of differentially methylated genes (DMGs). Scatter plots (**A**–**C**) show enriched pathways for all identified DMGs (**A**), hypermethylated DMGs (**B**), and hypomethylated DMGs (**C**), while plots (**D**–**F**) correspond to the analysis restricted to promoter-localized DMGs, depicting all (**D**), hypermethylated (**E**), and hypomethylated (**F**) promoter DMGs. In all panels, the vertical axis indicates pathway names and the horizontal axis represents the rich factor (with higher values denoting greater enrichment). The size of each point corresponds to the number of DMGs mapped to the respective pathway, and point color reflects the range of Q-values.

Our KEGG pathway analysis linked the differentially methylated genes (DMGs) to 282 distinct pathways. A comparative analysis between the *Mo*-infected and control groups (*p* < 0.01, Figure 7A) revealed significant enrichment in pathways related to immune and inflammatory responses. These included the B cell receptor signaling pathway, Fc epsilon RI signaling pathway, MAPK signaling pathway, Th17 cell differentiation, and the regulation of actin cytoskeleton.

The subtype analysis provided further details. Hypermethylated DMGs (Figure 7B) showed enrichment in several key immune signaling pathways, such as the B cell receptor and Fc epsilon RI signaling pathways, as well as the MAPK signaling pathway. We also observed enrichment for terms including the inflammatory mediator regulation of TRP channels and the oxytocin signaling pathway. While neuro-immune interactions are an area of interest, the direct biological relevance of these specific terms to pulmonary mycoplasmal infection requires further study and may be influenced by database annotation patterns. In contrast, hypomethylated DMGs (Figure 7C) were notably enriched in pathways directly related to pathogen defense and immune cell trafficking. These included Staphylococcus aureus infection, leukocyte transendothelial migration, and the Rap1 and MAPK signaling pathways.

A focused analysis on DMGs located within promoter regions identified 130 significantly enriched pathways (*p* < 0.01; Figure 7D). These were primarily involved in immune–inflammatory responses, metabolic processes, and cellular signaling. Key pathways included antigen processing and presentation, Th1 and Th2 cell differentiation, and the Notch signaling pathway. Promoter-specific hypermethylated DMGs (Figure 7E) were predominantly linked to immune regulation (e.g., antigen processing and presentation), metabolic processes (e.g., histidine metabolism), and cellular signaling (e.g., PI3K–Akt signaling pathway). Conversely, promoter-specific hypomethylated DMGs (Figure 7F) showed pronounced enrichment in infection-responsive immunity (e.g., NOD-like receptor signaling pathway) and specific metabolic pathways, alongside involvement in the MAPK signaling pathway.

In summary, the integrative analysis of genome-wide and promoter-specific DMGs demonstrates a convergent enrichment of core immune–inflammatory pathways. This pattern robustly aligns with the expected host response to a respiratory pathogen like Mycoplasma ovipneumoniae. The co-enrichment of certain neuro-related pathway terms among broader immune signatures was noted, but its functional significance in this specific disease context remains to be determined.

### 3.5. Verification of DMG Methylation Levels Using BSP

**Figure 8:**
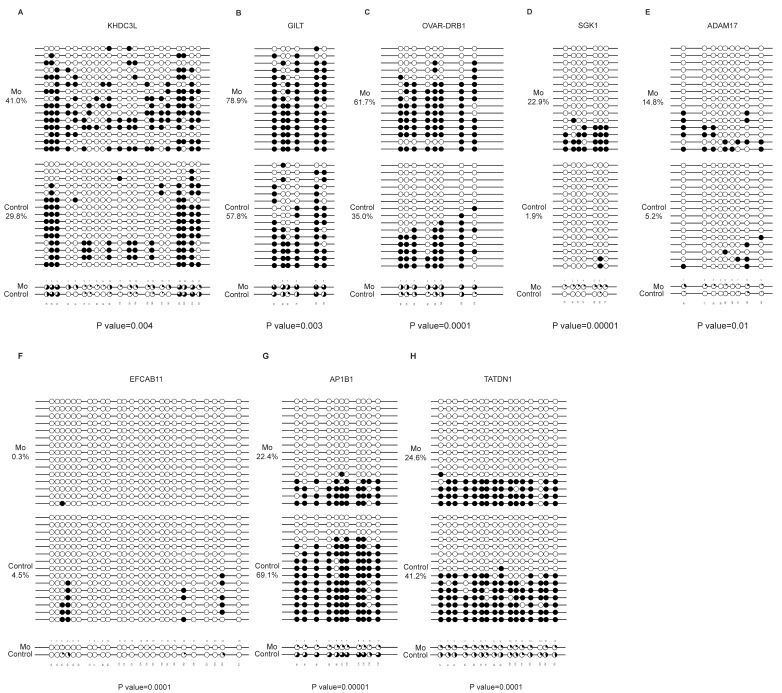
Bisulfite sequencing PCR (BSP) validation of differentially methylated genes (DMGs). (**A**–**E**) Validation of five hypermethylated DMGs (*KHDC3L*, *GILT*, *OVAR-DRB1*, *SGK1*, and *ADAM17*) selected from DMRs. (**F**–**H**) Validation of three hypomethylated DMGs (*EFCAB11*, *AP1B1*, and *TATDN1*) selected from DMRs. For each panel, methylation levels were confirmed by BSP, and sequencing results were analyzed using QUMA software. Each horizontal line represents a cloned sequence, with circles indicating individual CpG sites (white circles: unmethylated; black circles: methylated). Data are expressed as mean ± standard deviation from ≥3 biological replicates per group. Statistical significance was assessed using Fisher’s exact test.

Integration of the top 10 DMGs of hypermethylation and hypomethylation in RRBS (Table 2) with gene annotation identified eight DMGs (five hypermethylated and three hypomethylated) harboring significant DMRs within promoter areas for validating RRBS data. BSP analysis confirmed that the reliability of RRBS results for hypermethylated DMGs: *KHDC3L* methylation significantly increased from 29.8% in the control group to 41.0% in the *Mo*-infected group (Figure 8A), whereas that for *GILT* methylation rose from 57.8% to 78.9% (Figure 8B) and *OVAR-DRB1* from 35.0% to 61.7% (Figure 8C). Similarly, the data reliability for *SGK1* methylation elevated from 1.9% to 22.9% (Figure 8D), whereas that for *ADAM17* methylation increased from a basal level of 5.2% in the control group to 14.8% in the *Mo*-infected group (Figure 8E).

Furthermore, BSP validation confirmed concordance between the hypomethylated DMGs (*EFCAB11*, *AP1B1*, and *TATDN1*) and RRBS sequencing data. Specifically, the data reliability for *EFCAB11* methylation decreased to 0.3% in the *Mo*-infected group compared with 4.5% in the control group (Figure 8F); the reliability for *AP1B1* methylation declined to 22.4% in the *Mo*-infected group compared with 69.1% in the control group (Figure 8G); and the reliability for *TATDN1* methylation dropped to 24.6% in the *Mo*-infected group relative to 41.2% in the control group (Figure 8H). Collectively, these BSP results substantiated the reliability of the RRBS dataset.

### 3.6. MSP and QMSP-Based Validation of DMG Methylation

**Figure 9:**
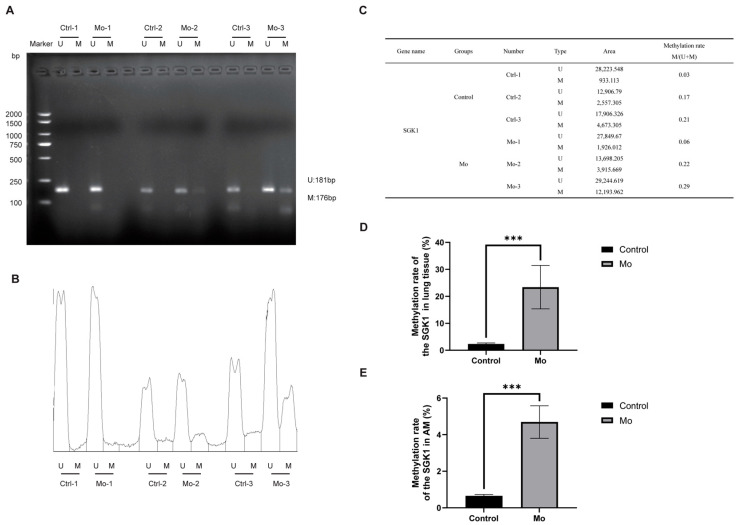
Validation of *SGK1* methylation by MSP and QMSP. (**A**) MSP gel electrophoresis of lung tissue samples for the *SGK1*. (**B**) Relative fluorescence intensity histogram of MSP amplicons from panel (**A**), quantified using ImageJ software. (**C**) Corresponding areas and calculated methylation proportions derived from the histogram in panel (**B**). (**D**) Methylation level of the *SGK1* in lung tissue measured by QMSP. (**E**) Methylation level of the *SGK1* in AMs measured by QMSP. Statistical significance between indicated groups determined using Student’s *t* test (*** *p* < 0.001).

**Figure 10:**
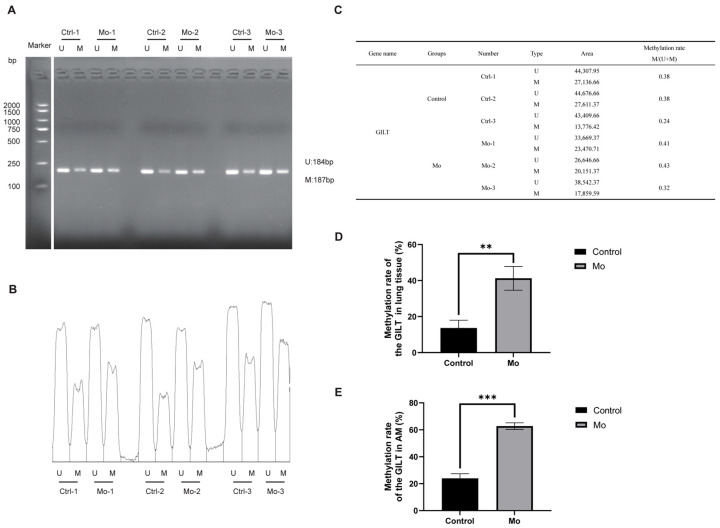
Validation of *GILT* methylation by MSP and QMSP. (**A**) MSP gel electrophoresis of lung tissue samples for the *GILT*. (**B**) Relative fluorescence intensity histogram of MSP amplicons from panel (**A**), quantified using ImageJ software. (**C**) Corresponding areas and calculated methylation proportions derived from the histogram in panel (**B**). (**D**) Methylation level of the *GILT* in lung tissue measured by QMSP. (**E**) Methylation level of the *GILT* in AMs measured by QMSP. Statistical significance between indicated groups determined using Student’s *t* test (** *p* < 0.01, *** *p* < 0.001).

Using primers targeting the *SGK1* and *GILT* genes, MSP amplification consistently yielded stronger band intensities in the *Mo*-infected group compared to the control group (Figure 9A and Figure 10A). Quantification of the gel fluorescence intensity with ImageJ software, presented as relative intensity histograms (Figure 9B and Figure 10B) and summarized in a combined table of band area and methylation proportion (Figure 9C and Figure 10C), confirmed this visual observation.

QMSP further validated these findings. The methylation level of the *SGK1* was significantly higher in the *Mo* group, measuring 23.40% in lung tissue (Figure 9D) and 4.69% in alveolar macrophages (AMs) (Figure 9E), compared to 2.35% and 0.65%, respectively, in the control group. Similarly, the *GILT* showed elevated methylation in the *Mo* group, with levels of 41.26% in lung tissue (Figure 10D) and 62.74% in AMs (Figure 10E), versus 13.70% and 23.94% in the control group.

Together, the MSP and QMSP results demonstrate that *Mo* infection induces increased methylation of the *SGK1* and *GILT* genes in both lung tissue and alveolar macrophages of hybrid argali sheep.

### 3.7. Analysis of Expression of Key DMGs Using RT qPCR

**Figure 11:**
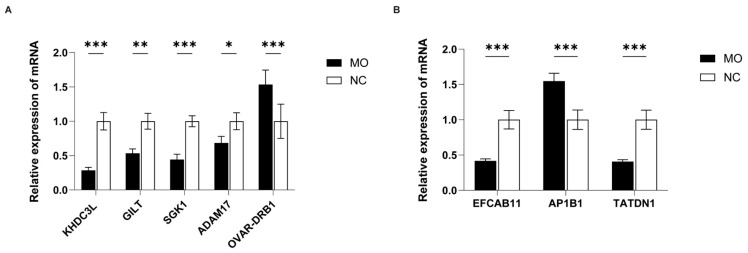
Relative mRNA expression of hyper- and hypomethylated DMGs in lung tissues across experimental groups. (**A**) Expression levels of hypermethylated DMGs. (**B**) Expression levels of hypomethylated DMGs. For both panels, the horizontal axis represents gene names, and the vertical axis indicates relative mRNA expression levels. Group differences are indicated by color. Data are expressed as mean ± standard deviation of ≥3 biological replicates. Statistical significance between indicated groups determined using Student’s *t* test (* *p* < 0.05, ** *p* < 0.01, *** *p* < 0.001).

RT-qPCR was performed to assess the relative mRNA expression levels of these eight genes in lung tissue samples. Among the hypermethylated genes, the relative expression levels of *KHDC3L* (0.28-fold), *GILT* (0.53-fold), *SGK1* (0.44-fold), and *ADAM17* (0.68-fold) decreased in the *Mo*-infected group compared with the control group (set as 1.00), consistent with high methylation levels in their promoter regions. However, the expression level of *OVAR-DRB1* increased (1.53-fold) (Figure 11A). Among the hypomethylated genes, the expression level of *AP1B1* increased (1.54-fold) in the *Mo*-infected group compared with the control group (1.00), corresponding to hypomethylation in its promoter region. In contrast, the expression levels of *EFCAB11* (0.41-fold) and *TATDN1* (0.40-fold) decreased (Figure 11B).

## 4. Discussion

DNA methylation is a fundamental epigenetic mechanism regulating gene expression and host responses to pathogenic infection. For instance, hepatitis B virus infection can induce host epigenetic reprogramming, thereby modulating antiviral immune gene expression [28]. However, the dynamic changes in DNA methylation triggered by Mycoplasma ovipneumoniae (*Mo*) infection in the lung tissue of argali hybrid sheep remain poorly characterized. This study employed high-throughput reduced representation bisulfite sequencing (RRBS) to generate a preliminary genome-wide DNA methylome map of infected sheep lung tissue, exploring the methylation remodeling associated with *Mo* infection.

Previous studies have utilized RRBS to investigate epigenetic regulatory mechanisms in Clostridium perfringens type C infection in piglets [29] and bovine respiratory disease [30]. In the present study, analysis of lung tissue from a limited cohort revealed that *Mo* infection was associated with a significant increase in genome-wide mCG methylation levels, suggesting the activation of epigenetic reprogramming centered on global hypermethylation. Methylation distribution analysis revealed the highest mCG levels in gene bodies and downstream 2 kb regions, and the lowest in promoter and 5′UTR regions, particularly near the transcription start site. This pattern is consistent with the general principles of mammalian genomic methylation [31], stemming from the presence of CpG islands (CGIs) in the promoter regions of most vertebrate genes [32,33]. CpG sites within CGIs are typically maintained in a hypomethylated state to support basal transcription, while those outside CGIs are more susceptible to methylation [34]. Indeed, promoter CGI methylation is frequently linked to transcriptional silencing [35,36], a trend consistent with the observed downregulation of most validated genes (*KHDC3L*, *GILT*, *SGK1*, and *ADAM17*) exhibiting promoter hypermethylation. It is important to note that the RRBS approach preferentially covers CpG-rich regions, and thus our map may not fully represent methylation dynamics in intergenic or low-CpG density regulatory elements that could also be functionally relevant during infection [37].

Alterations in promoter methylation are known to influence transcription factor binding efficiency [38]. Therefore, the analysis of differentially methylated genes (DMGs) within promoter regions is crucial for understanding potential methylation-mediated gene regulation. Gene Ontology (GO) and KEGG pathway enrichment analyses revealed that the most prominent and biologically coherent finding was the significant enrichment of both hyper- and hypomethylated DMGs in infection and immune–inflammatory pathways. These included antigen processing and presentation, B cell receptor signaling, Fc epsilon RI signaling, Th17 cell differentiation, and the MAPK signaling pathway. This robust enrichment pattern strongly aligns with the known role of *Mo* as a respiratory pathogen that elicits potent immune and inflammatory responses, which lends credibility to our dataset. We also observed enrichment of terms related to neural signaling among hypermethylated DMGs. Although neuro-immune interactions are recognized in other mucosal contexts [39], the direct biological relevance of these specific pathways to pulmonary mycoplasmosis remains unclear and may reflect, at least in part, limitations or inherent annotation biases in cross-species functional databases. Consequently, we interpret these findings with caution, considering them preliminary observations requiring independent validation and have focused our biological interpretation on the more directly pertinent immune pathways.

Using this dataset, we identified and validated several DMGs of particular interest. The hypermethylated genes *GILT* and *OVAR-DRB1* are key components of the antigen processing and presentation pathway (Figure S2). The altered methylation status of these genes correlated with changes in their expression, suggesting their potential role in dysregulated antigen presentation and inflammatory responses during *Mo* infection [40,41]. Functional enrichment analysis indicated that hypermethylated DMGs were significantly associated with broad regulatory processes. Notably, *SGK1*, a central component of the *PI3K–Akt* signaling pathway (Figure S4), exhibited hypermethylation and concurrent downregulation. Given its known role in modulating immune responses [42], our data suggest that *SGK1* may be part of the signaling network perturbed by *Mo* infection. Similarly, the hypermethylation and reduced expression of *ADAM17*, a sheddase involved in Notch and other immune signaling pathways (Figure S3) [43,44], may contribute to impaired immune regulation. This finding is consistent with previous reports demonstrating that *ADAM17* deficiency affects T cell homing and inflammatory resolution in lung infections [45]. Another hypermethylated gene, *KHDC3L*, has previously been associated with DNA damage and apoptosis [46]; its methylation changes may therefore influence cell fate decisions in infected tissue. Among hypomethylated genes, *EFCAB11* is involved in calcium binding [47], *AP1B1* has been linked to immune cell modulation (Figure S5) [48], and *TATDN1* plays a role in genome stability [49]. Their altered methylation states suggest broader functional perturbations extending beyond immediate immune pathways. To further validate these findings, we experimentally examined the methylation status of *SGK1* and *GILT*. We performed MSP and QMSP on bisulfite-converted DNA from lung tissue and alveolar macrophages (AMs). Consistent with the RRBS data, MSP revealed stronger amplification products in the *Mo*-infected group for both genes. Quantitative analysis by QMSP confirmed significantly elevated methylation levels of *SGK1* and *GILT* in infected lung tissue and, notably, also in AMs—a key immune cell type in the lung microenvironment. These results provide orthogonal validation of the methylation changes identified by high-throughput sequencing and underscore that epigenetic alterations occur not only in bulk lung tissue but also in resident immune cells, potentially amplifying local immune dysregulation.

The integrated use of BSP, MSP, and QMSP confirmed the reliability of RRBS-based methylation profiling. Moreover, correlation analysis between promoter methylation levels and corresponding gene expression (determined by RT-qPCR) revealed that methylation changes in most target genes corresponded to their expected expression patterns. Promoter-hypermethylated DMGs (*KHDC3L*, *GILT*, *SGK1*, and *ADAM17*) were downregulated, whereas the promoter-hypomethylated DMG (*AP1B1*) was upregulated, consistent with the established role of DNA methylation in transcriptional silencing [50,51,52]. However, notable exceptions were observed: the promoter-hypermethylated gene *OVAR-DRB1* showed increased transcription, while the promoter-hypomethylated genes *EFCAB11* and *TATDN1* exhibited decreased transcription. This finding underscores that transcriptional output is not governed solely by promoter methylation levels in a simple, linear, inverse manner. Rather, DNA methylation-mediated gene regulation is highly context-dependent and can be influenced by factors such as: (i) methylation changes at distal enhancers rather than promoters [53]; (ii) the combinatorial effects of other epigenetic marks, such as histone modifications [54]; and (iii) the altered cellular composition within the infected bulk lung tissue sample, in which methylation is assessed in one cell population while expression is measured in another could be measured simultaneously [55]. Future studies employing cell-type-specific or single-cell multi-omics approaches are needed to disentangle these complex regulatory layers [56,57].

Collectively, these intricate epigenetic reprogramming events correlate with the inflammatory phenotype induced by *Mo* infection. The observed genome-wide hypermethylation and immune gene alterations are consistent with the establishment of a persistent inflammatory microenvironment, potentially through the suppression of negative regulators or the activation of pro-inflammatory pathways. This notion is supported by previous findings demonstrating that *Mo* infection upregulates ovine respiratory pro-inflammatory mediators, including *IL-1β*, *IL-6*, and *NF-κB* [58]. Although we observed methylation changes in genes associated with neuro-related pathways, their functional significance in pulmonary mycoplasmosis remains speculative and warrants dedicated experimental inquiry.

## 5. Limitations and Future Perspectives

This study provides the first methylome map of *Mo*-infected sheep lungs, while the findings are robust and validated through multiple methods, we acknowledge certain limitations that inherently define this pioneering work and, importantly, open promising avenues for subsequent investigation. First, the modest sample size (*n* = 3 per group), while not uncommon in large-animal model studies employing deep sequencing, may limit the statistical power and generalizability of the findings. Therefore, our results should be considered a robust foundation for hypothesis generation. Second, our use of Reduced Representation Bisulfite Sequencing (RRBS), while effective for capturing CpG-dense regions, provides a limited view of the total methylome. Third, the analysis of bulk lung tissue, while providing a holistic overview of the pathological environment, inherently averages the methylation signals from diverse cell types. Finally, this study identifies robust correlations between DNA methylation and gene expression but does not establish causality. The next logical step is to move beyond correlation to functional validation. Future studies with larger, independent cohorts are essential to confirm the prevalence and variability of these methylation marks across the broader population. To resolve this heterogeneity, future work should leverage cutting-edge single-cell multi-omics technologies. Specifically, simultaneous single-cell RNA-seq and single-cell ATAC-seq or methyl-seq (e.g., scM&T-seq) would allow us to deconvolve the cellular complexity, The application of targeted epigenome-editing tools, such as CRISPR-dCas9 fused with DNA methyltransferases (for methylation) or demethylases (for demethylation), presents an exciting opportunity. By manipulating the methylation status of specific candidate genes and we may directly assess their functional impact of specific methylation changes. In conclusion, by addressing the limitations of this initial study through these proposed future directions, we can move from a descriptive understanding of the *Mo*-induced epigenome to a mechanistic and ultimately therapeutically actionable framework for combating this important respiratory disease.

## 6. Conclusions

This study presents a genome-wide DNA methylation profile of lung tissue from sheep infected with Mycoplasma ovipneumoniae, revealing an epigenetic reprogramming landscape dominated by widespread hypermethylation. We identified 3691 differentially methylated regions (DMRs) and validated the correlation between methylation status and expression changes for eight candidate genes, including *KHDC3L*, *GILT, OVAR-DRB1*, *SGK1*, and *ADAM17*. This work provides the first genome-wide methylome map associated with this infection in a ruminant host. The identified DMRs and key genes offer concrete targets for subsequent mechanistic investigation.

Collectively, these findings furnish foundational data for elucidating the host’s epigenetic regulatory response to infection. The DMRs may serve as candidate references for developing infection-related epigenetic biomarkers. Furthermore, the aberrant methylation states of certain key genes, such as *SGK1* and *GILT*, suggest potential entry points for future exploration of adjuvant therapies targeting epigenetic modifications. Overall, this methylome atlas establishes a data resource and provides leads for further research into infection–host epigenetic interactions in this economically important animal disease model.

## Figures and Tables

**Table 1:** Sequencing data quality control chart by Reduced Representation Bisulfite Sequencing (RRBS).

Sample Name	Raw Reads	Raw Bases (G)	Clean Reads	Clean Bases (G)	Clean_Ratio (%)	Q20 (%)	Q30 (%)	GC Content (%)	BS Conversion Rate (%)
*Mo* 1	35,024,223	10.51	34,370,652	8.41	80.02	97.53	93.25	34.23	99.593
*Mo * 2	33,559,529	10.07	32,940,558	8.05	79.94	97.54	93.18	34.36	99.58
*Mo * 3	33,414,356	10.02	32,776,512	7.98	79.64	97.53	93.23	34.29	99.588
Control 1	37,252,137	11.18	36,504,000	8.86	79.25	97.46	92.96	34.38	99.586
Control 2	33,506,836	10.05	32,657,063	7.73	76.92	96.93	91.74	34.32	99.595
Control 3	34,358,234	10.31	33,342,228	7.86	76.24	96.65	91.07	34.3	99.615

**Table 2:** Top 10 DMGs of hypermethylation and hypomethylation in RRBS.

Methylation Type	DMGs	Mo_meanMethy	Control_meanMethy	Diff.Methy	AreaStat	Region
hypermethylated	KHDC3L	0.545556	0.225863	0.319693	124.17544	promoter
	GILT	0.719975	0.502249	0.217726	22.33915	promoter
	SGK1	0.213841	0.063678	0.150163	30.56868	promoter
	ADAM17	0.361424	0.1282	0.233225	67.22678	promoter
	OVAR-DRB1	0.640741	0.327233	0.313508	43.13307	promoter
	CPLX1	0.909972	0.210135	0.699837	92.49984	promoter
	TMEM184A	0.785986	0.27447	0.511516	33.10031	promoter
	Tgif1	0.56883	0.283577	0.285253	42.89791	promoter
	Pdk3	0.913188	0.149084	0.764104	11.29362	promoter
	Msantd1	0.874081	0.10646	0.767621	14.46684	promoter
hypomethylated	EFCAB11	0.026445283	0.315370351	−0.28893	−46.25334	promoter
	AP1B1	0.338341548	0.609884684	−0.27154	−48.98554	promoter
	TATDN1	0.169331849	0.368861816	−0.19953	−44.73395	promoter
	CIBAR2	0.245963358	0.867807716	−0.62184	−11.68999	promoter
	NUP107	0.139455371	0.740561193	−0.60111	−11.16543	promoter
	TMEM184A	0.228266077	0.789417855	−0.56115	−16.81358	promoter
	INCA1	0.232659854	0.728960494	−0.4963	−10.55955	promoter
	IGLV5−45	0.05039301	0.367218889	−0.31683	−37.34895	promoter
	DGCR2	0.329639587	0.610777674	−0.28114	−11.79547	promoter
	PNMA8B	0.080487422	0.451025391	−0.37054	−17.30565	promoter

## Data Availability

The original contributions presented in this study are included in the article/supplementary material. Further inquiries can be directed to the corresponding authors.

## Supplementary Materials

The following supporting information can be downloaded at: https://www.mdpi.com/article/10.3390/microorganisms14030597/s1. Supplementary Table S1. Primers used for BSP and MSP in this study. Supplementary Table S2. Primers used for RT-qPCR and QMSP in this study. Figure S1. GO hierarchical annotation of all DMGs. (A) Hypermethylated DMGs. (B) Hypomethylated DMGs. The horizontal axis represents enriched GO terms, and the vertical axis indicates the number and percentage of genes annotated to the term (including child terms). Figure S2. KEGG pathway enrichment analysis of hypermethylated DMGs-promoter. The red squares denote genes enriched in the “antigen processing and presentation” pathway. Figure S3. KEGG pathway enrichment analysis of hypermethylated DMGs-promoter. The red squares denote genes enriched in the “PI3K–Akt signaling pathway”. Figure S4. KEGG pathway enrichment analysis of hypermethylated DMGs-promoter. The red squares denote genes enriched in the “Notch signaling pathway”. Figure S5. KEGG pathway enrichment analysis of hypomethylated DMGs-promoter. The red squares denote genes enriched in the “human immunodeficiency virus 1 infection” pathway.

## Abbreviations

Ams	Alveolar macrophages
BSP	Bisulfite sequencing PCR
DMRs	Differentially Methylated Regions
DMLs	Differentially Methylated Loci
DMGs	Differentially methylated genes
DMGs-promoter	Differentially methylated genes of promoter
DPI	Days Post-Inoculation
DSS	Dispersion Shrinkage for Sequencing data
MSP	Methylation-specific PCR
QMSP	Quantitative methylation-specific PCR
GO	Gene Ontology
KEGG	Kyoto Encyclopedia of Genes and Genomes
*Mo*	*Mycoplasma ovipneumoniae*
RRBS	Reduced Representation Bisulfite Sequencing
TE	Transposable Elements
