# Supplementary material for: Construction of a DNA Methylation Map of Argali Hybrid Sheep During Mo Infection
Source: Microorganisms. 2026 Mar 6;14(3):597. doi: 10.3390/microorganisms14030597 (PMC13028814; doi:10.3390/microorganisms14030597)
Supplement: Supplementary file 1 [file microorganisms-14-00597-s001.zip › microorganisms-4164624-supplementary.pdf]

Supplementary table S1. Primers used for BSP and MSP in this study

Primer Name	Primer sequence (5'to 3')
KHDC3L-Forward	TGAAGTAGGGTTTGTGATATATATT
KHDC3L-Reverse	CACCTAAAAACCTAACTAATAAAAAC
GILT-Forward	GGTTAGGATAGTGGGATGGGTTTT
GILT-Reverse	CACTAACCAATACATAATAATCCC
GILT-MSP-U-Forward	AATTGTGATTTTTGAAAGATTAGTTG
GILT-MSP-U- Reverse	ACTCCTAACTTCCTTACCAACACT
GILT-MSP-M-Forward	AATTGTGATTTTCGAAAGATTAGTC
GILT-MSP-M- Reverse	TATACTCCTAACTTCCTTACCGACG
SGK1-Forward	GATTTTAGAAAGTAAAGGAGGGAGAGG
SGK1-Reverse	CATCAAACTACAACTCCCAAACATCT
SGK1-MSP-U-Forward	AATTGTGATTTTTGAAAGATTAGTTG
SGK1-MSP-U-Reverse	CCTAACTTCCTTACCAACACTAACAC
SGK1-MSP-M-Forward	AATTGTGATTTTCGAAAGATTAGTC
SGK1-MSP-M-Reverse	CTTCCTTACCGACGCTAACG
ADAM17-Forward	GAGATTTTTTTGGAGAAGGGAATG
ADAM17-Reverse	CCACTAAACCCCAAAATTCTACAACT
OVAR-DRB1-Forward	GGGAGAGAGAGAGATAGATAGAGATAGATA
OVAR-DRB1-Reverse	CACAAAAACCCCAACTTCTACTTAC
EFCAB11-Forward	TTTATTTATTTTTTGTGTTTTGAGGGT
EFCAB11-Reverse	CCCATAAACTATTCCAAAAAAAC
AP1B1-Forward	GTTAGTGTGATGAGTTATTTTTGTTAAGGA
AP1B1-Reverse	CTCAAAATCAACTTCTAAAAACTATAAAAAA
TATDN1-Forward	GTAAGGTTTTTTAGATGTGGG
TATDN1-Reverse	CAATAAAAAATAAAAACCCAAAATAC

Supplementary table S2. Primers used for RT-qPCR and QMSP in this study

Primer Name	Primer sequence (5'to 3')
KHDC3L-Forward	CGAGGCTGAAATCTTGATA
KHDC3L-Reverse	GCTGACGGTGATAGTTAG
GILT-Forward	CCTAGAGGAAATGAATGACA
GILT-Reverse	CTAGCACACTCCATGATG
GILT-qMSP-U-Forward	AATTGTGATTTTTGAAAGATTAGTTG
GILT-qMSP-U- Reverse	ACTCCTAACTTCCTTACCAACACT
GILT-qMSP-M-Forward	AATTGTGATTTTCGAAAGATTAGTC
GILT-qMSP-M- Reverse	TATACTCCTAACTTCCTTACCGACG
SGK1-Forward	TTCTCCTGGCAAGACACAA
SGK1-Reverse	AACATTCCGCTCCGACATAA
SGK1-qMSP-U-Forward	AATTGTGATTTTTGAAAGATTAGTTG
SGK1-qMSP-U-Reverse	CCTAACTTCCTTACCAACACTAACAC
SGK1-qMSP-M-Forward	AATTGTGATTTTCGAAAGATTAGTC
SGK1-qMSP-M-Reverse	CTTCCTTACCGACGCTAACG
ADAM17-Forward	CATTCTCAAGTCTCCACAA
ADAM17-Reverse	TAGCAACATCTTCACATCC
OVAR-DRB1-Forward	AGAGCAAGATGATGAGTG
OVAR-DRB1-Reverse	TCCTGAAGTAGATGAAGAG
EFCAB11-Forward	GCTGATTCTGTGATGTCT
EFCAB11-Reverse	GTCTTACTTCATTGCGATAC
AP1B1-Forward	TACTTCACCACGACTAAG
AP1B1-Reverse	CCTTCTTCTTCTCCTTCT
TATDN1-Forward	TGGAGTTGCTGAATCTTG
TATDN1-Reverse	GCTGAGTATCTTTGGAACA
HSP70-Forward	GCCAAATCGTTCAATAAATC
HSP70-Reverse	CGTCCAATAATAAAACATCAC
Sheep β-actin- Forward	GCGGCATTCACGAAACTA
Sheep β-actin- Reverse	TGTTGGCGTAGAGGTCTT

Sup Fig S1.

A

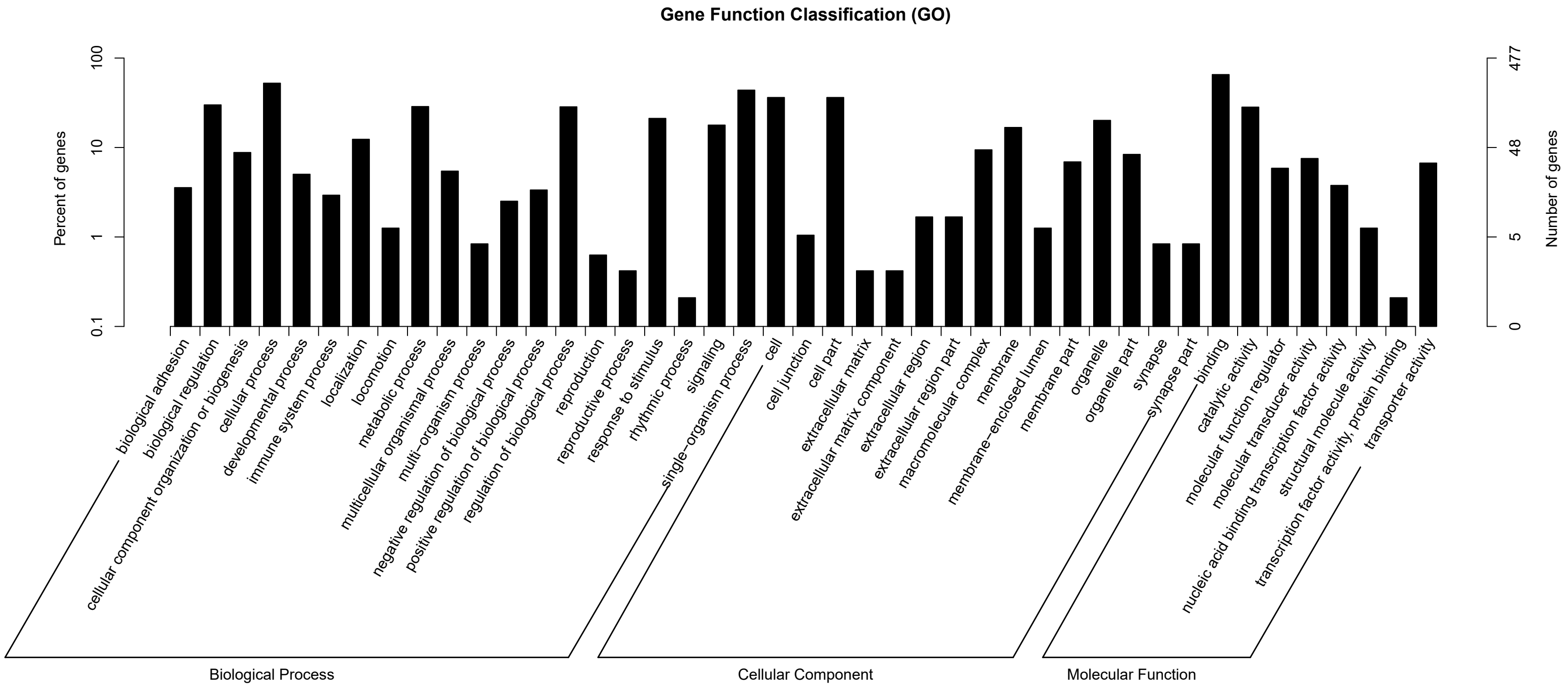
Gene Function Classification (GO)
Percent of genes
100
10
1
0.1
Number of genes
477
48
5
0
biological adhesion
biological regulation
cellular component organization or biogenesis
cellular process
developmental process
immune system process
localization
locomotion
metabolic process
multicellular organismal process
multi−organism process
negative regulation of biological process
positive regulation of biological process
regulation of biological process
reproduction
reproductive process
response to stimulus
rhythmic process
signaling
single−organism process
cell
cell junction
cell part
extracellular matrix
extracellular matrix component
extracellular region
extracellular region part
macromolecular complex
membrane
membrane−enclosed lumen
membrane part
organelle
organelle part
synapse
synapse part
binding
catalytic activity
molecular function regulator
molecular transducer activity
nucleic acid binding transcription factor activity
structural molecule activity
transcription factor activity, protein binding
transporter activity
Biological Process
Cellular Component
Molecular Function

B

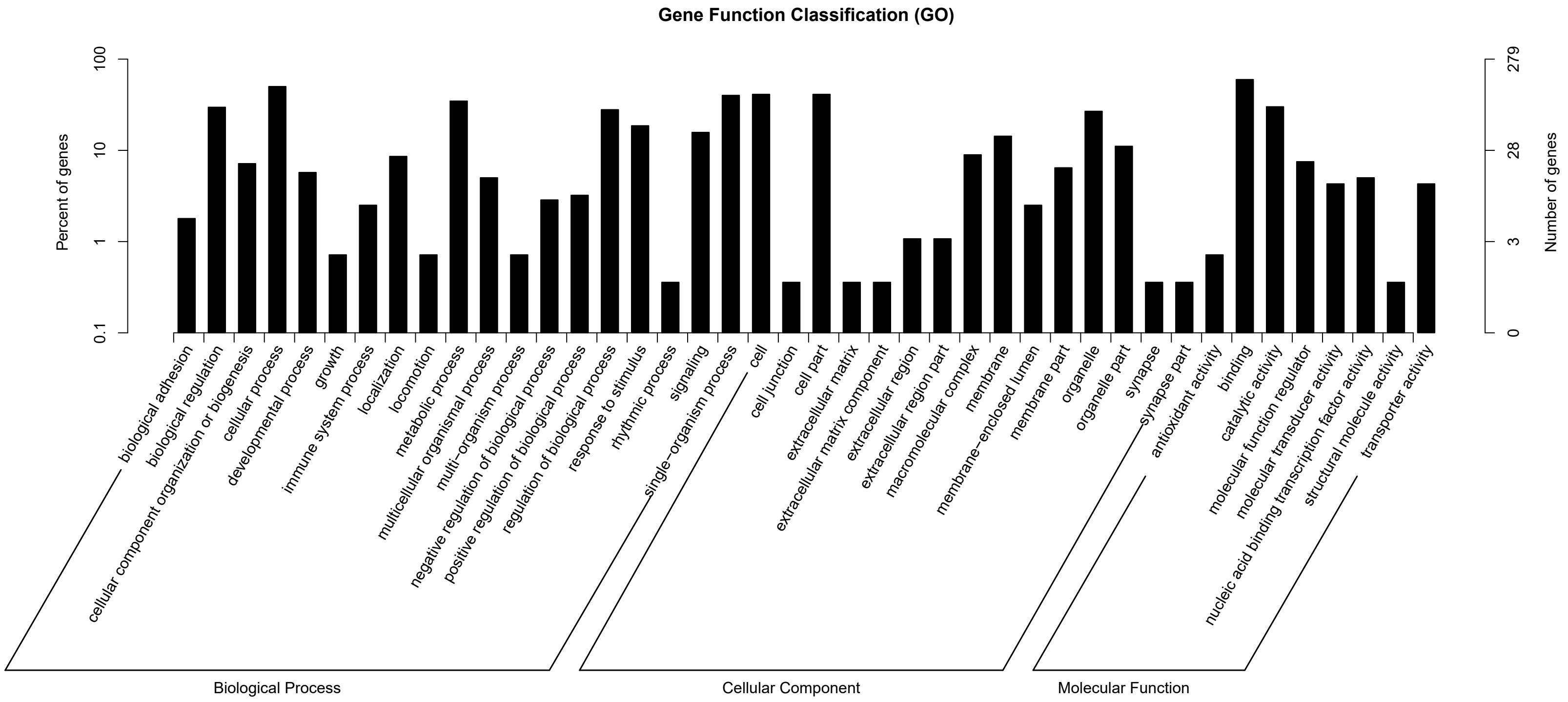
Gene Function Classification (GO)
Percent of genes
100
10
1
0.1
Number of genes
279
28
3
0
biological adhesion
biological regulation
cellular component organization or biogenesis
cellular process
developmental process
growth
immune system process
localization
locomotion
metabolic process
multicellular organismal process
multi−organism process
negative regulation of biological process
positive regulation of biological process
regulation of biological process
response to stimulus
rhythmic process
signaling
single−organism process
cell
cell junction
cell part
extracellular matrix
extracellular matrix component
extracellular region
extracellular region part
macromolecular complex
membrane
membrane−enclosed lumen
membrane part
organelle
organelle part
synapse
synapse part
antioxidant activity
binding
catalytic activity
molecular function regulator
molecular transducer activity
nucleic acid binding transcription factor activity
structural molecule activity
transporter activity
Biological Process
Cellular Component
Molecular Function

Sup Fig S2.

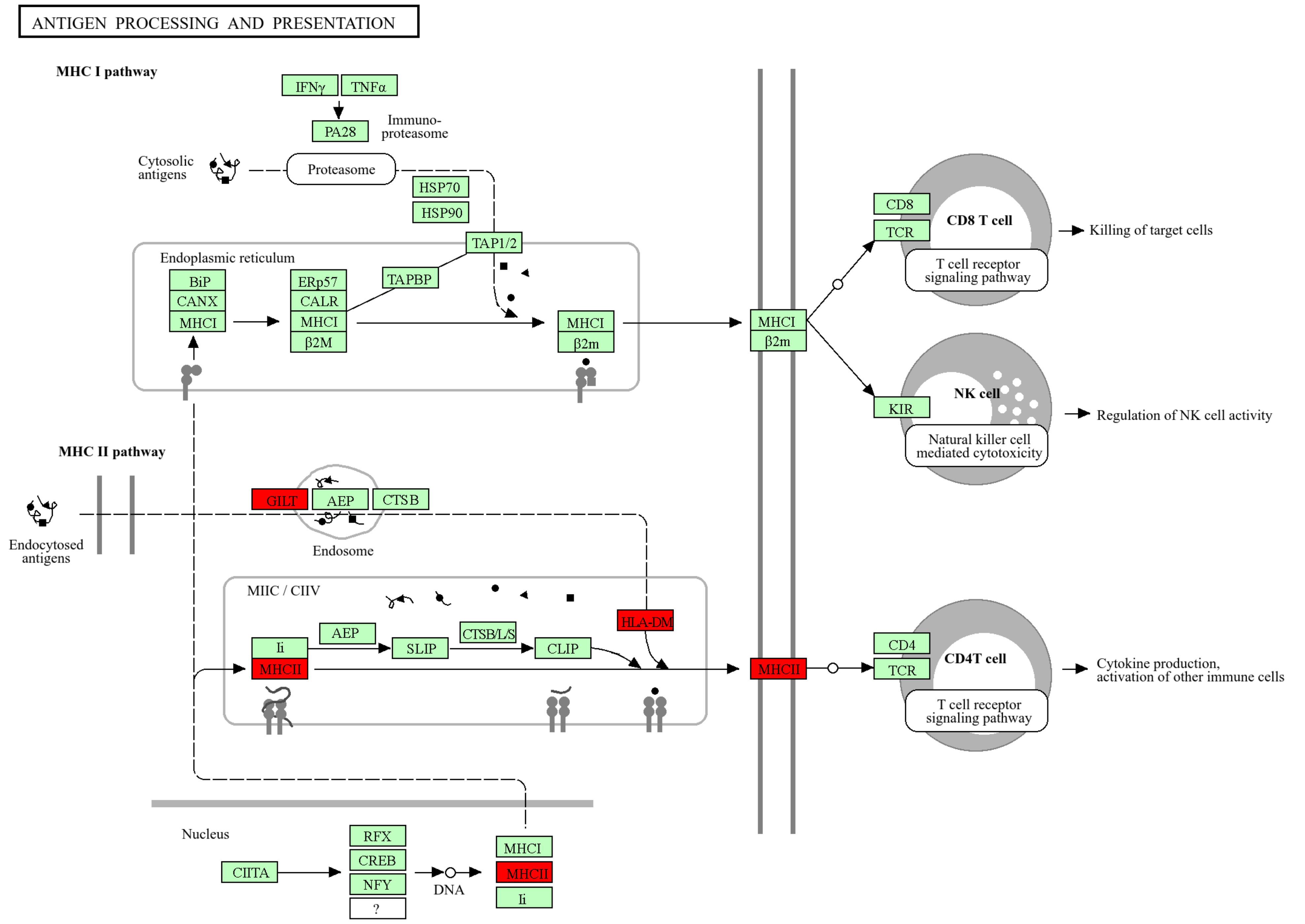

04612 6/26/23
(c) Kanehisa Laboratories

Sup Fig S3.

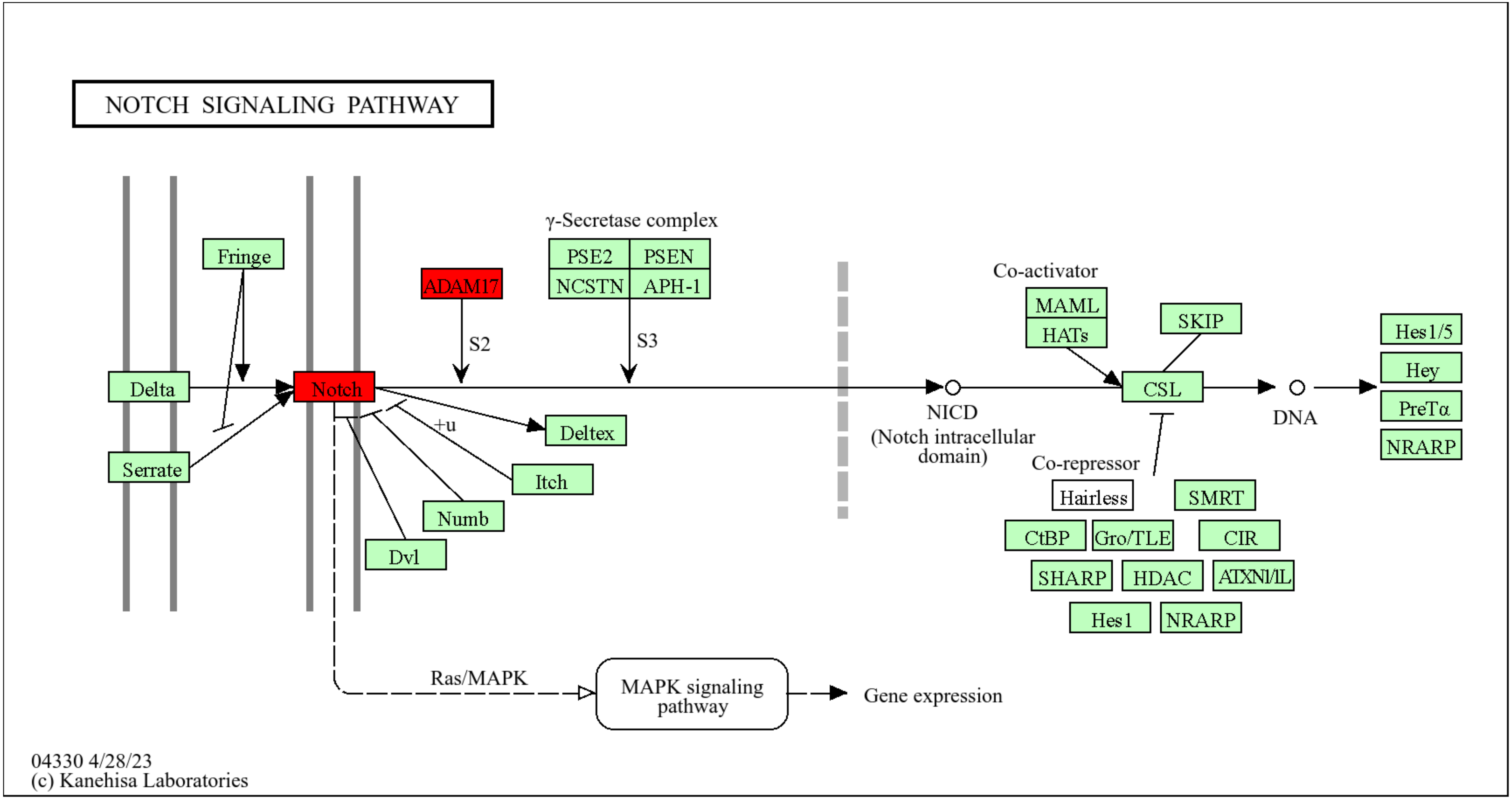
NOTCH SIGNALING PATHWAY
Fringe
Delta
Serrate
Notch
ADAM17
S2
γ-Secretase complex
PSE2
PSEN
NCSTN
APH-1
S3
+u
Deltex
Itch
Numb
Dvl
NICD
(Notch intracellular domain)
Co-activator
MAML
HATs
SKIP
CSL
DNA
Hes1/5
Hey
PreTα
NRARP
Co-repressor
Hairless
SMRT
CtBP
Gro/TLE
CIR
SHARP
HDAC
ATXN1/1L
Hes1
NRARP
Ras/MAPK
MAPK signaling pathway
Gene expression
04330 4/28/23
(c) Kanehisa Laboratories

Sup Fig S4.

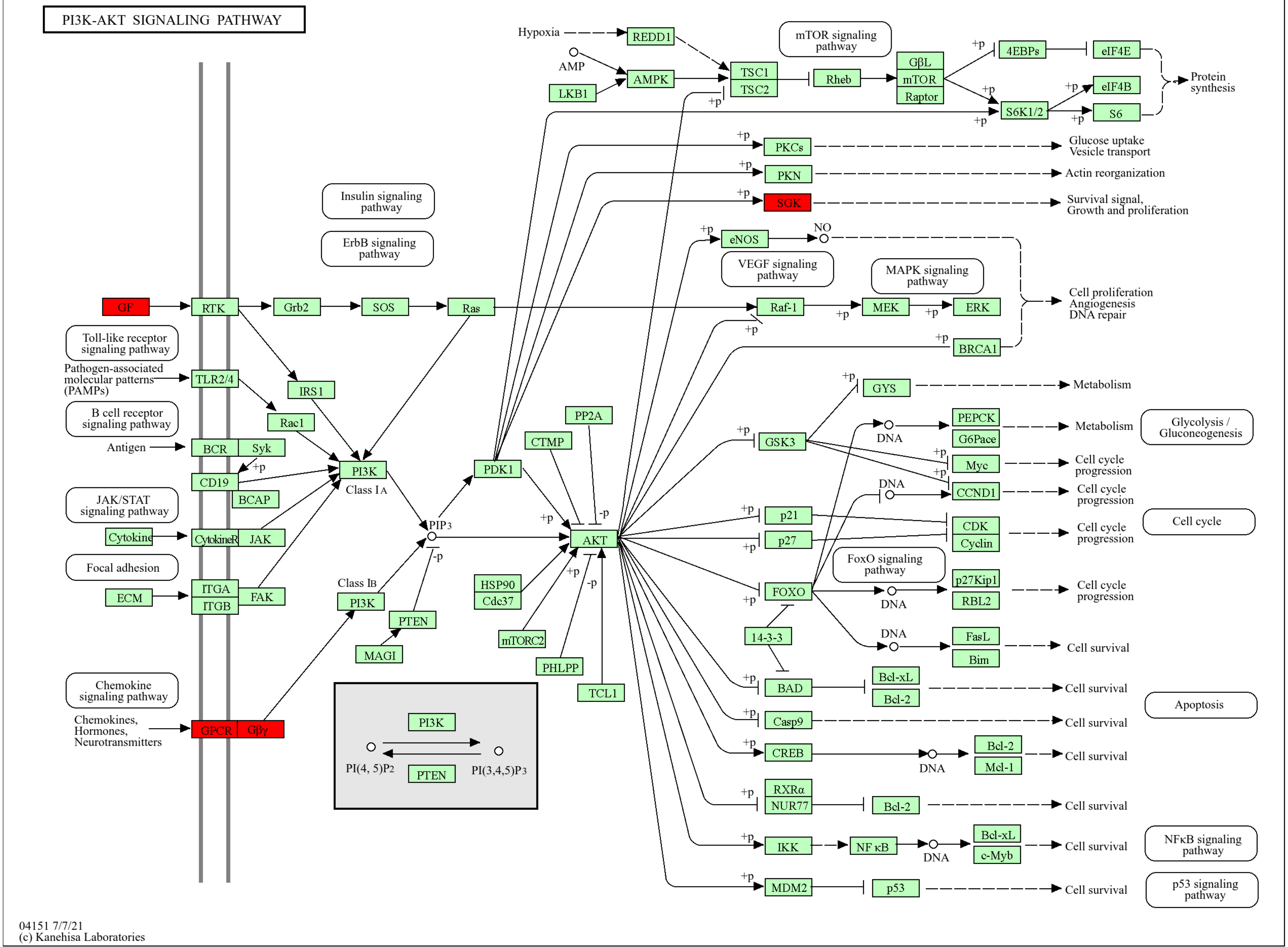
PI3K-AKT SIGNALING PATHWAY
Hypoxia
REDD1
AMP
AMPK
LKB1
TSC1
TSC2
mTOR signaling pathway
Rheb
GβL
mTOR
Raptor
4EBPs
eIF4E
eIF4B
S6K1/2
S6
Protein synthesis
PKCs
Glucose uptake
Vesicle transport
PKN
Actin reorganization
SGK
Survival signal, Growth and proliferation
Insulin signaling pathway
ErbB signaling pathway
eNOS
NO
VEGF signaling pathway
MAPK signaling pathway
GF
RTK
Grb2
SOS
Ras
Raf-1
MEK
ERK
Cell proliferation
Angiogenesis
DNA repair
BRCA1
Toll-like receptor signaling pathway
Pathogen-associated molecular patterns (PAMPs)
TLR2/4
IRS1
Rac1
GYS
Metabolism
B cell receptor signaling pathway
Antigen
BCR
Syk
CD19
BCAP
PI3K
Class IA
PDK1
CTMP
PP2A
GSK3
PEPCK
G6Pace
Glycolysis / Gluconeogenesis
Myc
Cell cycle progression
CCND1
JAK/STAT signaling pathway
Cytokine
CytokineR
JAK
PIP3
AKT
p21
p27
CDK
Cyclin
Cell cycle
Focal adhesion
ECM
ITGA
ITGB
FAK
Class IB
PI3K
PTEN
MAGI
HSP90
Cdc37
mTORC2
PHLPP
TCL1
FoxO signaling pathway
FOXO
p27Kip1
RBL2
14-3-3
FasL
Bim
Cell survival
BAD
Bcl-xL
Bcl-2
Apoptosis
Chemokine signaling pathway
Chemokines, Hormones, Neurotransmitters
GPCR
Gβγ
PI3K
PI(4, 5)P2
PTEN
PI(3,4,5)P3
Casp9
CREB
Mcl-1
RXRα
NUR77
IKK
NF κB
c-Myb
NFκB signaling pathway
MDM2
p53
p53 signaling pathway
DNA
+p
-p
04151 7/7/21
(c) Kanehisa Laboratories

Sup Fig S5.

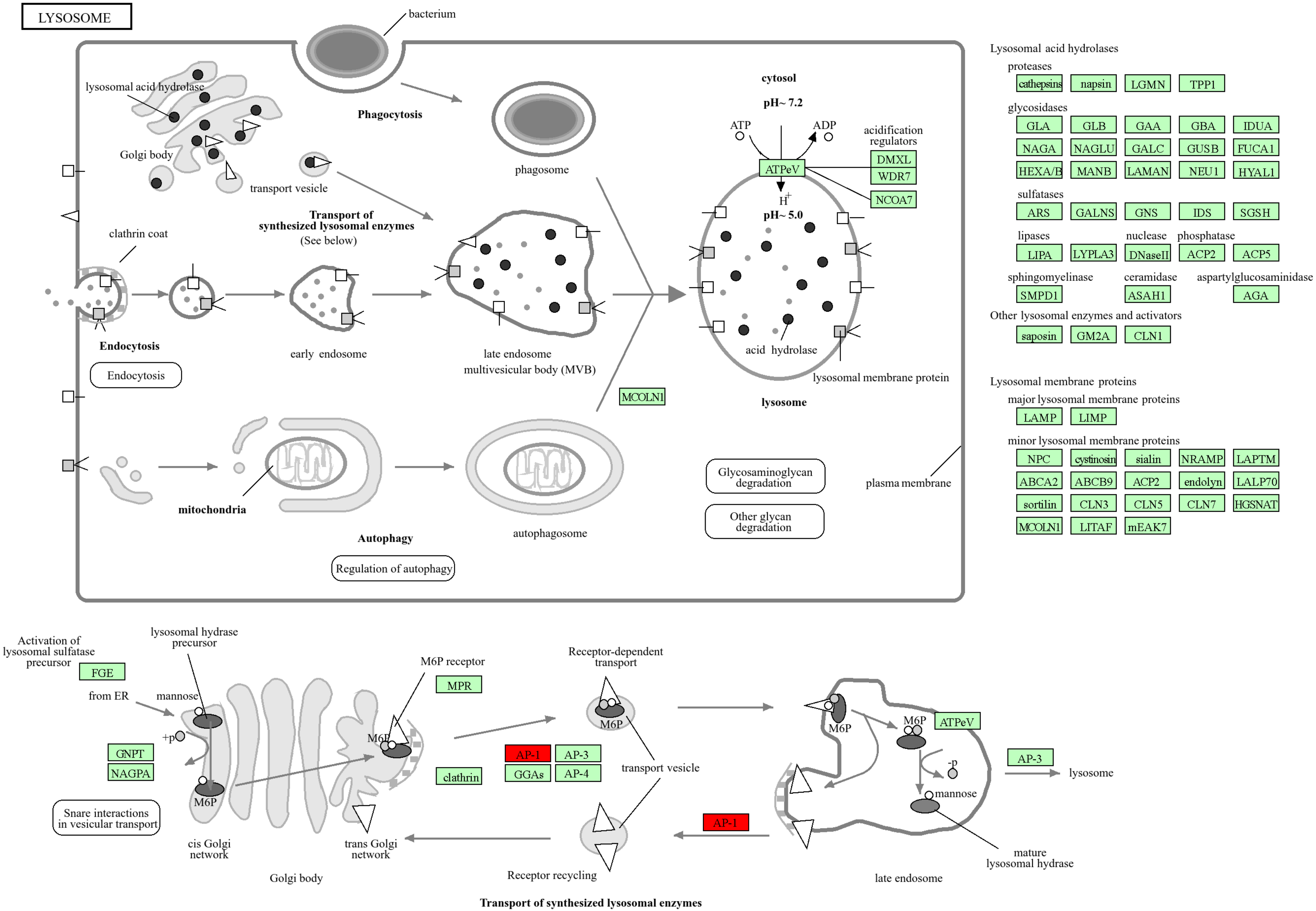
LYSOSOME
bacterium
lysosomal acid hydrolase
Golgi body
transport vesicle
Phagocytosis
phagosome
cytosol
pH~ 7.2
ATP
ADP
ATPeV
acidification regulators
DMXL
WDR7
NCOA7
pH~ 5.0
acid hydrolase
lysosomal membrane protein
lysosome
Transport of synthesized lysosomal enzymes (See below)
clathrin coat
Endocytosis
Endocytosis
early endosome
late endosome multivesicular body (MVB)
MCOLN1
mitochondria
Autophagy
autophagosome
Regulation of autophagy
Glycosaminoglycan degradation
Other glycan degradation
plasma membrane
Lysosomal acid hydrolases
proteases
cathepsins
napsin
LGMN
TPP1
glycosidases
GLA
GLB
GAA
GBA
IDUA
NAGA
NAGLU
GALC
GUSB
FUCA1
HEXA/B
MANB
LAMAN
NEU1
HYAL1
sulfatases
ARS
GALNS
GNS
IDS
SGSH
lipases
LIPA
LYPLA3
nuclease
DNaseII
phosphatase
ACP2
ACP5
sphingomyelinase
SMPD1
ceramidase
ASAH1
aspartylglucosaminidase
AGA
Other lysosomal enzymes and activators
saposin
GM2A
CLN1
Lysosomal membrane proteins
major lysosomal membrane proteins
LAMP
LIMP
minor lysosomal membrane proteins
NPC
cystinosin
sialin
NRAMP
LAPTM
ABCA2
ABCB9
ACP2
endolyn
LALP70
sortilin
CLN3
CLN5
CLN7
HGSNAT
MCOLN1
LITAF
mEAK7
Activation of lysosomal sulfatase precursor
FGE
lysosomal hydrase precursor
from ER
mannose
+p
GNPT
NAGPA
M6P
Snare interactions in vesicular transport
cis Golgi network
trans Golgi network
Golgi body
M6P receptor
MPR
M6P
clathrin
AP-1
AP-3
GGAs
AP-4
Receptor-dependent transport
M6P
transport vesicle
Receptor recycling
AP-1
M6P
M6P
ATPeV
-p
mannose
mature lysosomal hydrase
late endosome
AP-3
lysosome
Transport of synthesized lysosomal enzymes
